# Prevalence and predictors of musculoskeletal pain among Danish fishermen – results from a cross-sectional survey

**DOI:** 10.1186/s12995-016-0140-7

**Published:** 2016-11-15

**Authors:** Gabriele Berg-Beckhoff, Helle Østergaard, Jørgen Riis Jepsen

**Affiliations:** 1 Unit for Health Promotion Research, University of Southern Denmark, Niels Bohrs Vej 9, 6700 Esbjerg, Denmark; 2 Centre of Maritime Health and Society, University of Southern Denmark, Niels Bohrs Vej 9, Esbjerg, 6700 Denmark

**Keywords:** Fishermen, Musculoskeletal pain, Work load, Cross-sectional study

## Abstract

**Background:**

Fishermen work in a physically challenging work environment. The aim of this analysis was to estimate the prevalence and predictors of musculoskeletal pain among Danish fishermen.

**Method:**

A cross-sectional survey in a random sample of Danish fishermen was done with application of the Nordic questionnaire regarding musculoskeletal pain considering lower back, shoulders, hand neck, knee, upper back elbow, hip and feet. In total, 270 fishermen participated in the study (response rate: 28%). Workload, vessel type, skipper, duration of work, sideline occupation, days/weeks of fishing at sea, age, BMI and education were used as predictors for the overall musculoskeletal pain score (multiple linear regression) and for each single pain site (﻿multinomial logistic regression﻿).

**Results:**

The prevalence of pain was high for all musculoskeletal locations. Overall, more than 80% of the responding Danish fishermen reported low back pain, which in 37% lasted for a minimum of 30 days during the past year. In the multiple linear regression analysis, middle workload was associated with a 32% (95% CI: 19-46%) and high workload with 60% (95% CI: 46-73%) increased musculoskeletal pain score compared to low work load. Multinomial logistic regression models showed that workload was the only predictor for all pain sites, in particular regarding upper and lower limb pain.

**Conclusion:**

Although changes were implemented to improve the fishermen’s work environment, the work continues to be physically demanding and impacting their musculoskeletal pain. Potential explanation for this unexpected result like increased work pressure and reduced financial attractiveness in small scale commercial fishery needs to be confirmed in future research.

## What this article adds

Fishermen work in a challenging physical work environment and therefore have a high prevalence of musculoskeletal pain. However, during the last 10 to 20 years, several positive structural changes for the physical work environment took place, but it does not lead to a decreased musculoskeletal pain in fishermen’s. The only stringent predictor for this pain is still the workload.

## Background

Physical workload of commercial fishing has been highly reduced in contemporary fishing in countries like Denmark, but may still be excessive at times depending on the type of fishing and the applied gear. Not only do fishermen work in challenging settings, their work has little routine and is dictated by various external factors such as weather and waves [1, 2]. Fishery is regarded as a dangerous occupation as demonstrated by a high rate of occupational fatalities [3] and of hospitalisation for various diseases [4–6]. Furthermore, though the work environment may still include physical demands, little is known about the current prevalence of musculoskeletal pain in fishermen [7]. During the last two decades, many new safety measures in Danish fisheries have been developed and applied, and consequently positive structural changes for the physical work environment in fisheries have taken place. There has been development in the used equipment, and it has facilitated the ergonomic loads, new vessels with better technique have been developed and introduced, and information of correct moving and carrying heavy loads has been implemented [8, 9]. However, the most cited publication on the prevalence of low back pain in fishermen derives from an old cross-sectional study of 1642 Swedish deep-sea fishermen in which approximately 50% experienced low back symptoms, their most common impairment, during the last 12 months [10]. In a cohort study started in 1999 on 215 fishermen in North Carolina, USA, low back pain was also the most frequent symptom with a prevalence of 52% [11]. Seven percent of 210 fishermen in a British cross-sectional study from 2007 had visited a doctor because of low back pain during the past year [12]. A survey in Turkish Aegean small scale fishermen reports that, between 2009 and 2010, 84% of respondents had musculoskeletal disorders leading to physician visits within one year [13]. Finally, a recent cross sectional study from Sri Lanka done in 2011 revealed a prevalence of musculoskeletal symptoms of 61%; prevalence of low back pain was 37.6% [14]. In a comprehensive literature research further recent studies estimating musculoskeletal pain in fishermen were not found. None of the studies discussed the population based approach to omit selection bias about potential job related pain.

Predictors for musculoskeletal pain in fishermen are rarely studied. In the North-Carolina cohort study [1, 11], the following predictors for low back pain were tested: age, smoking year’s length of time in occupation, type of fishing and gear, job title, and fishing part time, years of fishing experience, or working more than one job. Significant increased risks for low back pain could be shown for young age (18–29) and history of low back pain. The authors additionally stated that job characteristics for low back pain are undetermined [1, 11].

Therefore the aim of this analysis is to estimate prevalence and predictors of musculoskeletal pain in Danish fishermen considering a population based approach. Due to the structural changes that have taken place in the trade and the reduced workload in contemporary fishery, our expectation was a reduced prevalence of musculoskeletal pain.

## Method

This cross-sectional survey took place between February and April 2015 and addressed 2500 randomly selected active fishermen registered with the Danish AgriFish Agency [15]. The study was promoted through a leaflet and press release and questionnaires were mailed out to potential respondents. To increase participation rate, the questionnaire could be completed either online (SurveyXact), or on paper and returned by mail, and an additional reminder was sent to non-respondents. Developed procedures were guided by the STROBE statement [16]. Informed consent was obtained from participants and this study has been approved by the Danish data Protection Agency (Jnr. 2014-41-3245).

**Fig. 1:**
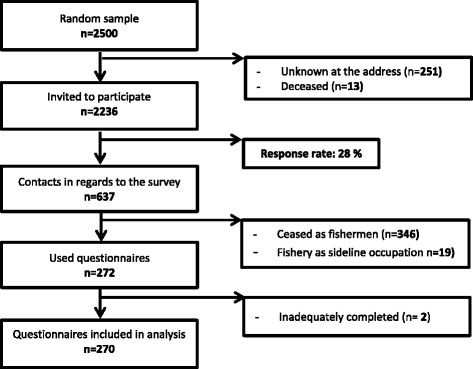
Flowchart for the selection of the study population

Of the selected fishermen, 251 had an unknown address and 13 had died. Out of the remaining 2,236 fishermen, 637 answered the questionnaire resulting in a response rate of 28%. However, out of the responding fishermen, 355 were no longer full time active fishermen. Two fishermen were additionally deleted due to many missing answers. Finally, 270 fishermen were used for analyses. The questionnaire collected general health data and socio-demographic information as well as work-related information (see Fig. 1).

Based on the Nordic musculoskeletal questionnaire [17], questions on *musculoskeletal pain* during the past 12 months were posed for the following anatomical sites: Neck, shoulder, elbow, hand, upper back, lower back, hip, knee, and foot. Potential categories for the Danish version was 0 days, 1–7 days, 8 to 30 days, 31 to 90 days, more than 90 days, and every day [18]. For descriptive purposes, pain lasting for a minimum of 30 days was presented. For an additional pain score summing up the pain in all nine anatomic sites, a conditional missing imputation was used with answers missing for up to four questions. Missing answers were calculated out of the mean value of the remaining answers from the relevant person. To estimate the consistency of the overall score given the 9 different musculoskeletal pain sites, Cronbach alpha was used. It was 0.91 indicating a very good internal consistency of the scale [19].

The *physical workload* was estimated by questions developed during the FINALE project [20] addressing the frequency of seven tasks: standing, pushing and pulling, carrying and lifting, lifting with hands above shoulders, bending forwards with the back, twisting and bending, and heavy work with fingers. Participants answers were coded (5) full time; (4) ¾ of time; (3) half of time; (2) a quarter of time; (1) rarely; and (0) never. For analysis, a score was developed by the sum of all seven questions. A conditional missing imputation was used with up to two missing answers. Missing answers were calculated out of the mean value of the remaining answers from the relevant person. Cronbach alpha was 0.81 indicating a good internal consistency of the scale [18]. For categorical purposes, tertils were built described as low, middle, or high workload.

Questions about work-related information reflected differences between *skipper* and *deckhands* for the analysis. In order not to omit missings (*n* = 19), we regarded these persons as deckhands, because skippers would always know their tasks while deckhands might have difficulties in defining their jobs. For the question about the *type of vessel* used, we considered the most frequently used type of vessel. The following types of vessels were presented: trawlers, Danish seiners, netters, liners, potters, and “others”. The relevant missing answers (*n* = 5) were rated as “others”. Information on days/weeks of fishing *at sea* was categorised into “1 day”, “up to one week”, and “more than one week”. More specific categorisation for longer stay at sea wasn’t feasible due to the small numbers of fishermen sailing longer time. The *duration of work* (in years) and *side line occupation* (yes or no) were also reported.

Demographic variables included the fishermen’s *age* and *education* (basic, skilled, and advanced). Based on self-reported weight (kg) and height (m), body mass index (*BMI)* was calculated (kg/m^2^) and categorized as normal (<25 kg/m^2^), overweight (25 – 30 kg/m^2^) and obese (>30 kg/m^2^) [21].

*Time to response* was applied for checking potential selection bias with regard to pain. The completed questionnaires were collected time-dependently. For the internet version, the time for completion of the questionnaire was automatically saved while for the printed version, the date of receipt was used. The fastest completed questionnaire was received one day after distribution while the latest arrived 70 days later. For analysis purposes, this time-variable was split into tertiles (early, middle, and late responders).

Statistical analyses were conducted in SAS Version 9.4 (*P* < 0.05). The prevalence of pain at different locations was presented overall and stratified for early, middle, and late responders to check for a potential selection bias related to musculoskeletal pain (data not shown). The association between workload (as score) and pain (low, middle and high workload) was analyzed by multiple linear regression. Considered predictors were work task, boat type, years of fishing, duration of work experience, days/weeks fishing at sea, sideline occupation, education, and BMI. Model assumptions were graphically tested and fulfilled after log transformation of the pain score.

A multinomial logistic regression model compared different pain locations within pain duration of 1 to 30 days, of more than 30 days, and no pain, given the last group as reference group. The proportional OR was used derived using a multinomial logistic regression model. We expect that a logistic regression gives trustful estimates and standard errors compared for example to the log binomial model. Estimates or standard errors do not differ between the models when high outcome prevalence’s are considered [22]. In contrast to the conditional imputation for musculoskeletal pain score, in categorical variables such a procedure is not feasible any more. Instead, an additional category “missing” was considered in the multinomial logistic regression to keep persons with missing values in the analysis. The models were adjusted for workload, vessel type, years of fishing, duration of work experience, days/weeks fishing at sea, sideline occupation, education, and BMI.

## Results

Table 1 shows the work characteristics and demographics of the study population. All responding fishermen were males and most were skippers and worked on trawlers. Fishing voyages were mostly short with half of the fishermen at sea for one day only. Most fishermen had a basic education, were overweight, and above 50 years of age.

**Table 1:** Work characteristics and socio demographic variable cross sectional survey of the 270 Fishermen in 2015

	Number	Percent	Missing
Skipper	167	66.53	0^a^
Boat			
Trawler	118	43.70	0^b^
Danish seiners	14	5.19	
Netters and liners	75	27.28	
Potters	28	10.37	
Other type	35	12.96	
Days/weeks of fishing on sea			
1 day	124	48.06	12
>1 to 7 days	90	34.88	
More than 7 days	44	17.05	
Education			
Basic education	137	54.80	8
Skilled worker	63	25.20	
Advanced education	50	20.00	
BMI			
Normal weight (<25 kg/m^2^)	55	21.57	3
Overweight (25 – 30 kg/m^2^)	119	46.67	
Obese (≥30 kg/m^2^)	81	31.76	
Age			
<30 years	20	7.81	3
30-40 years	21	8.20	
40-50 years	52	20.31	
50-60 years	82	32.03	
≥ 60 years	81	31.64	
Total	270	100.00	

^a^ *n* = 19; included in deckhands

^b^ *n* = 5; included in other type

The prevalence of musculoskeletal pain at different locations distributed is presented in Table 2. Pain lasting less than 30 days during the last year and more than 30 days per year was chosen to explore weak and more prolonged musculoskeletal pain, respectively. Overall, any low back and shoulder pain were most common and experienced by 4/5 of the fishermen during the last year (pain ≤ 30 days per year and pain > 30 days per year together). Hand and neck pain was secondly most frequent with a pain prevalence of around 2/3. Prolonged low back, shoulder and hand pain was also common and present in about 1/3 of the fishermen. The pain prevalence in all musculoskeletal locations did not differ between early, medium and late respondents (data not shown).

**Table 2:** Prevalence of less and more than 30 day per year pain in different areas of the body; cross sectional survey in 270 fishermen 2015

	Number	Percent
Lower Back		
No pain	42	17.9
Pain ≤30 days/year	104	44.3
Pain >30 days/year	89	37.9
missing	35	
Hand		
No pain	80	34.9
Pain ≤30 days/year	87	38.0
Pain >30 days/year	62	27.1
missing	41	
Knee		
No pain	86	39.6
Pain ≤30 days/year	88	40.6
Pain >30 days/year	43	19.8
missing	53	
Hip		
No pain	144	56.9
Pain ≤30 days/year	74	29.3
Pain >30 days/year	35	13.8
missing	17	
Elbow		
No pain	131	53.0
Pain ≤30 days/year	85	34.4
Pain >30 days/year	31	12.6
missing	23	
Shoulder		
No pain	48	21.4
Pain ≤30 days/year	104	46.4
Pain >30 days/year	72	32.1
missing	46	
Neck		
No pain	89	33.6
Pain ≤30 days/year	112	42.3
Pain >30 days/year	64	24.2
missing	5	
Upper back		
No pain	102	41.6
Pain ≤30 days/year	95	38.8
Pain >30 days/year	48	19.6
missing	25	
Feet		
No pain	147	61.8
Pain ≤30 days/year	59	24.8
Pain >30 days/year	32	13.5
missing	32	

Multiple linear regression analyses of predictors for the overall pain score showed that fishermen categorized with middle workload had a 32% increased musculoskeletal pain score compared to the fishermen with low of workload, whereas fishermen with high workload had a 60% increased pain score compared to workers with low work load. These associations were highly significant and showed a positive trend. Having a sideline occupation was associated with a 15% significantly reduced musculoskeletal pain score. All other considered variables such as skipper, boat type, days/weeks fishing at sea, education, BMI, and duration of work had no effect on pain after additional adjustment for workload (Table 3).

**Table 3:** Multiple linear regression model on predictors on the overall musculoskeletal pain score. Cross sectional survey in 270 fishermen, 2015

	Musculoskeletal pain score	
	Beta^a^	95% CI
Workload:		
Low workload	**Ref.**	
Middle workload	**0.32**	**0.19-0.46**
High workload	**0.60**	**0.46-0.73**
BMI [in kg/m^2^]	−0.00	−0.01; 0.01
Duration of work experience [in years]	0.00	−0.00; 0.00
Sideline occupation		
Yes	**−0.15**	**−0.28; −0.02**
No	ref.	
Occupation:		
Captain	0.05	−0.06; 0.17
Other than captain	Ref.	
Vessel type		
Trawler	0.03	−0.09 ;0.15
Other than trawler	Ref.	
Education		
More than basic education	0.06	−0.19; 0.03
Basic education or less	Ref.	
Days/weeks of fishing at sea		
1 day	Ref.	
1 to 7 days	0.06	−0.06;0.19
More than 7 days	0.07	−0.09;0.24

^a^bold estimates are significant

Table 4 shows the multinomial logistic regression models for the different pain locations using the same predictors as in the multiple linear model. The only constant predictor for all pain sites was workload which indicated that all locations of prolonged musculoskeletal pain were highly significantly associated with high workload displaying odds ratios mostly exceeding 10. High workload was particularly associated with prolonged pain in hands and feet (odds ratios around 20 and more). High workload was already consistently and significantly associated with pain of less than 30 days of duration in elbow, hand and upper back. However, the concrete estimates need to be interpret with caution due to the wide confidence intervals particular for the group with musculoskeletal pain for more than 30 days per year. There were two additional predictors; sideline occupation was associated with less shoulder pain, and work duration of more than 30 days per year was a risk factor for hip pain.

**Table 4:** Multinomial logistic regression^a^ of predictors on musculoskeletal pain sites – only significant results are shown; Cross sectional survey in 270 fishermen, 2015

	No pain^b^	Pain less than 30 days/years	Pain more than 30 days/years	Missing
	OR	OR^c^ (95% CI)	OR^c^ (95% CI)	OR^c^ (95% CI)
Model 1: Lower back				
Workload				
Low workload	1 (ref)			
Middle workload		0.91 (0.34-2.48)	**4.64 (1.48-14.57)**	0.85 (0.24-3.03)
High workload		0.99 (0.33-2.99)	**12.46 (3.70-41.99)**	1.40 (0.37-5.23)
Model 2: Shoulder				
Workload				
Low workload	1 (ref)			
Middle workload		1.13 (0.45-2.87)	2.88 (0.94-8.80)	0.90 (0.31-2.67)
High workload		2.66 (0.89-7.96)	**11.99 (3.41-42.06)**	1.40 (0.39-5.01)
Sideline occupation				
No	1 (ref)			
Yes		1.32 (0.51-3.43)	**0.28 (0.08-0.96)**	1.13 (0.37-3.45)
Model 3: Hand				
Workload				
Low workload	1 (ref)			
Middle workload		**3.06 (1.28-7.30)**	**6.70 (2.12-21.17)**	1.09 (0.38-3.08)
High workload		**4.06 (1.61-10.25)**	**19.82 (6.26-62.79)**	0.86 (0.25-2.94)
Model 4: Neck				
Workload				
Low workload	1 (ref)			
Middle workload		1.09 (0.51-2.35)	**3.87 (1.37-10.90)**	nn.
High workload		1.89 (0.82-4.22)	**10.18 (3.52-29.40)**	0.69 (0.03-18.57)
Model 5: Knee				
Workload				
Low workload	1 (ref)			
Middle workload		2.01 (0.87-4.66)	**4.24 (1.26-14.33)**	0.76 (0.29-2.01)
High workload		**4.46 (1.85-10.73)**	**10.99 (3.18-38.02)**	1.46 (0.53-4.01)
Model 6: Upper back				
Workload				
Low workload	1 (ref)			
Middle workload		**2.83 (1.28-6.25)**	**37.35 (4.20-332.10)**	3.01 (0.81; 11.19)
High workload		**5.16 (2.14-12.46)**	n.n.	2.61 (0.58-11.78)
Model 7: Hip				
Workload				
Low workload	1 (ref)			
Middle workload		0.97 (0.43-2.22)	**3.17 (0.86-11.67)**	3.07 (0.63-14.96)
High workload		**2.93 (1.37-6.25)**	**9.21 (2.62-32.37)**	1.34 (0.20-8.92)
Work duration				
per year		1.01 (0.99-1.04)	**1.05 (1.01-1.09)**	1.03 (0.98-1.08)
Model 8: Feet				
Workload				
Low workload	1 (ref)			
Middle workload		2.13 (0.88-5.12)	**19.43 (2.30-164.59)**	1.64 (0.53-5.08)
High workload		**2.50 (1.04-5.97)**	**24.00 (2.90-199.01)**	1.20 (0.39-3.73)
Model 9: Elbow				
Workload				
Low workload	1 (ref)			
Middle workload		**2.23 (1.00-4.97)**	**3.42 (0.88-13.26)**	0.99 (0.25; 3.95)
High workload		**5.18 (2.26-11.84)**	**10.98 (2.88-41.81)**	1.18 (0.29-4.92)

^a^All models contained workload, vessel type, years of fishing, duration of work experience, sideline occupation, education, and BMI.

^b^no pain during the last year

^c^bold estimate are significant, as the 95% CI does not include 1

## Discussion

In this cross sectional study about fishermen the prevalence of musculoskeletal pain is high and the main predictor of musculoskeletal pain is the workload of fishermen which continues to be physically demanding in Danish fishermen even though structural changes took place. With more than 80% of the Danish fishermen reporting only low back pain during the past year and 37% reporting low back pain for at least 30 days during the past year, the prevalence of low back pain among Danish fishermen is high. A more recent survey among Turkish Aegean small-scale fishermen (2009–2010) found a similarly high prevalence of musculoskeletal disorders of 84%, even though the prevalence is based on physician visits within one year and combined different forms of musculoskeletal disorders [13]. Older surveys using the same questionnaire in Sweden (10, 1988) and USA (11, 1999) showed 50% and 51% of respondents claiming low back pain during the last year, respectively, which are both lower than the prevalence found in this current survey. However, direct comparison with these surveys is hampered as the Danish version of this questionnaire uses one additional category (the category of 30 to 90 days of pain) [18]. A greater choice of options for answers with more categories may lead to higher rates of prevalence. Furthermore, the recent Danish fishermen had the highest mean age compared to the other studies’ participants dealing with musculoskeletal pain. However, with regard to the musculoskeletal pain, no trend between age and pain could be identified which was similar to other surveys observing this association [10, 11].

Population based surveys in occupational settings may be hampered by low response rates which can additionally bias prevalence estimations. With a response rate of 75.5%, the Swedish survey is optimal and therefore the most cited, though it is the oldest [10]. Response rate in the British survey was 68% [12]. The response rate of the cross sectional survey in Sri Lanka was named to be unbelievable 100% [14]. No information on the response rate was given in the cohort study from USA [11, 23] and in the Turkish survey [13]. The response rate of 28% in the current survey is low, but this is to be expected in a population based postal survey of fishermen. To check for potential selection bias, we collected time to response and compared prevalence’s between early, middle and late respondents. For such a bias the expectation was that participants with pain would answer earlier due to a higher interest in the study than participants without pain [24]. The absence of such trend suggests that pain prevalence rates from our survey are not systematically over or under presented and they might represent musculoskeletal pain of Danish fishermen. In summary, age and low response rate cannot explain the high pain prevalence in the Danish fishermen study. Conclusively, lack of decrease in musculoskeletal pain prevalence and the high rates of reported pain in Danish fishermen were unexpected.

In the present study, workload was the most important predictor for musculoskeletal pain. For all pain locations there was a consistent, significant, and pronounced association between high workload and pain exceeding 30 days in the last year even though the exact estimates should be considered carefully due to the wide confidence intervals. The association between workload and pain was most pronounced for the extremities such as hands and feet. Additionally, the overall multiple linear analyses as well as the multinomial logistic regression on shoulder pain demonstrated that sideline occupation was significantly negatively associated with pain indicating that having a sideline occupation was associated with less pain. The American survey revealed the same result even though not significant [11]. This can be explained in various ways. Firstly, fishermen with a sideline occupation may be less exposed to the physically challenging work environment as full-time fisherman and therefore experience less musculoskeletal pain. Secondly, the healthiest fishermen may have the capacity to take extra work in addition to fishing. Further research is necessary to clarify underlying causal pathways.

When adjusting for workload neither occupational tasks, boat type, education, BMI, work experience nor time on sea were associated with musculoskeletal pain. Similar findings were reported in the American survey in which, with the exception of gender, there was no significant association between low back pain and age, BMI, fishing full time or having an additional job [11].

A number of health and safety measures on board the vessels have been developed and introduced, and structural changes in the trade have taken place in recent years [8, 9]. In spite of this there is no trend of improved musculoskeletal health in the studied sample of fishermen. What are the potential reasons for this? Firstly, globalisation, overcapacity and reduced fish prices has reduced the number of fishing vessels as well as fishermen in Western countries including Denmark [8, 25]. Fishing quota and season limit days restricts the catch, the volume of which commercial fishery is highly dependent. Therefore the fishermen strive to fish as much as possible within the limits indicated by increasing the workload and work pace at sea. A further explanation might be the reduced job satisfaction over time compared with the high work satisfaction of fishermen, which has been discussed as reasons for former potential underreporting of musculoskeletal symptoms in Swedish fishermen [2, 10, 26]. Furthermore, it is shown recently that job-dissatisfaction increase in Danish fishermen nowadays [27]. Current structural changes in Western fishery with high competition, overcapacity, and job uncertainty might lead to dissatisfaction, unhealthy behaviour and decreased health. Fishermen were previously satisfied with their job; they are now under pressure, particularly with regard to the consequences of globalisation, the highly regulated trade, and the reduced financial attractiveness of small scale commercial fisheries. Unfortunately, no similar survey was done in Denmark before structural changes took place, and therefore direct comparisons are hampered and suggestions are still speculative. Future research should focus on the health outcome of the potentially enhancing effect of occupational stress on tremendous workload in fishermen. Finally, the age structure is changing. Job uncertainty and unclear future occupational perspectives result in concerns of young workers to start a career as fishermen. Consequently, the majority of fishermen are quite old.

The very good internal consistency of the scale with a Cronbach alpha of 0.91 showed that all locations of pain are highly inter-correlated. A fisherman reporting hand pain is likely to also report pain in other locations. However, as shown, workload is the best predictor for pain, particularly in extremities, like hands and feet, which should therefore be addressed in further research. This can be confirmed by recommendations of the fishermen who were asked for suggestions relating to workplace improvements on board. Sixteen fishermen provided their ideas relating to standing (more space, rubber mats, better footwear, and chairs), as well as monotony and lifting (cutting table, lifting equipment, conveyor, and other mechanical equipment to reduce manual loads).

The cross-sectional study design causes some limitations. The direction of the association between the predictor and pain cannot be ascertained. Although the questions regarding exposure addressed the current circumstances, it cannot be ruled out tha t previous exposures explain a proportion of the reported pain. Additionally fishermen suffering from pain may experience more pain with certain current exposures such as workload, which may therefore be overrated. Furthermore, a healthy worker selection may cause bias because fishermen with pain are more likely to have left the trade rather than fishermen without pain and consequently those who remain in the occupation are healthier [28]. These limitations are innate in the cross-sectional design and cannot be overcome. While most studies undertaken in this context have used the same design, future research should apply longitudinal designs to allow a better estimation of a causal pathway of the relevant associations. The low response rate (28%) is a well-known limitation in representative occupational surveys. It was shown that a potential selection bias due to low response rate with regard to musculoskeletal pain was not likely. Another limitation is the missing values in pain-related questions, some of which were due to an error in the questionnaire construction. Missing values were considered through conditional missing imputation in the pain score and for the single pain sites, no such imputation was done. However, an additional category for missing values was considered to see if missing by itself has an effect on the outcome. Overall, the odds ratios for missing categories were small and far from significance. These stringent results in all analyses suggest that missing values do not dramatically bias the overall analyses.

## Conclusion

In conclusion, the prevalence of musculoskeletal pain is high in the studied sample of Danish fishermen and pain in all considered locations is related to the perceived workload. Future research should focus more on the causes for such a high prevalence. To consider healthy worker bias and to follow up with the overall life time occupation in fishermen, a cohort design would allow for consideration of the relative importance in the development of musculoskeletal pain of various exposures as well as different reasons for working full-time, part-time, or quitting the job. Further interventions should particularly focus on the effect of workload on pain in hands and feet.

The work as a fisherman remains physically demanding although much less than it has been prior to the implementation of structural changes. The potential effect of working under pressure due to consequences of globalisation, the highly regulated trade, and the reduced financial attractiveness of small scale commercial fisheries require future research that focus on the health outcome of the potentially enhancing effect of occupational stress on tremendous workload in fishermen.

## Abbreviations

95% CI: 95% Confidence interval
BMI: Body mass index
Nn: not named
OR: Odds ratio
Ref: reference
